# Age Is Important for the Early-Stage Detection of Breast Cancer on Both Transcriptomic and Methylomic Biomarkers

**DOI:** 10.3389/fgene.2019.00212

**Published:** 2019-03-26

**Authors:** Xin Feng, Jialiang Li, Han Li, Hang Chen, Fei Li, Quewang Liu, Zhu-Hong You, Fengfeng Zhou

**Affiliations:** ^1^BioKnow Health Informatics Lab, College of Computer Science and Technology, Jilin University, Changchun, China; ^2^Key Laboratory of Symbolic Computation and Knowledge Engineering of Ministry of Education, Jilin University, Changchun, China; ^3^BioKnow Health Informatics Lab, College of Software, Jilin University, Changchun, China; ^4^Key Laboratory of Symbolic Computation and Knowledge Engineering of Ministry of Education, Jilin University, Changchun, China; ^5^Xinjiang Technical Institute of Physics and Chemistry, Chinese Academy of Sciences, Ürümqi, China

**Keywords:** age, feature selection, TriVote, BRCA, classification, transcriptome, methylome

## Abstract

Patients at different ages have different rates of cell development and metabolisms. As a result, age should be an essential part of how a disease diagnosis model is trained and optimized. Unfortunately, most of the existing studies have not taken age into account. This study demonstrated that disease diagnosis models could be improved by merely applying individual models for patients of different age groups. Both transcriptomes and methylomes of the TCGA breast cancer dataset (TCGA-BRCA) were utilized for the analysis procedure of feature selection and classification. Our experimental data strongly suggested that disease diagnosis modeling should integrate patient age into the whole experimental design.

## Introduction

Some types of cancers grow faster in younger hosts. Renal cancer has an average growth rate of 0.3 cm per year and many clinical studies focused on the surveillance of small tumors only in elderly patients (Mues et al., 2010; Mehrazin et al., 2014). However, renal cancers in younger patients may grow at a much larger rate of 2.13 cm per year (Gofrit et al., 2015), which requires more frequent follow-up examinations. Prostate cancer was mostly diagnosed at an older age (>65 years old), but the early-onset cases (<55 years old) had a much faster growth rate and a stronger genetic association (Salinas et al., 2014).

Breast cancer has the largest incidence rates for females in both China (Chen et al., 2016) and United States (Siegel et al., 2018) and tends to grow faster in younger females (Weedon-Fekjaer et al., 2008). One of twenty breast tumors may double in diameter from 10 mm within 1.2 months, compared with 6.3 years for the same proportion with the slowest growth rates (Weedon-Fekjaer et al., 2008). Generally, younger age was one of the risk factors for poor prognosis and high aggressiveness (Bardia and Hurvitz, 2018; Lee et al., 2018). Even the genomic or transcriptomic biomarkers demonstrated different associations with younger breast cancer patients compared to older ones (Wang et al., 2018) and required age-specific treatments (Kim et al., 2018).

Breast cancer diagnosed at its early stage may be treated with mastectomy or lumpectomy and systematically reduces relapse risk (Kummerow et al., 2015; Santa-Maria et al., 2015). Early-stage breast cancer was usually diagnosed by radiological imaging technologies (Simos et al., 2014) or molecular biomarkers (Duffy et al., 2015). X–ray-based mammogram (Kashyap et al., 2017; Sinthia and Malathi, 2018) and breast magnetic resonance imaging (MRI) were the predominant choices for detecting the candidate lesion sites of breast cancer (Wang et al., 2013; Loggers et al., 2016). Serum microRNA and urine DNA damage were also recently observed to have strong associations with early-stage breast cancer (Guo et al., 2017; An et al., 2018). Unfortunately, these early-stage breast cancer detection technologies did not integrate the age information in the decision-making process.

This study hypothesized that the integration of age information may improve the performance of the biomarker detection problem, which is known as the feature selection problem in the machine learning area (Alshawaqfeh et al., 2017; Xu et al., 2018). Following this, we split the transcriptomic and methylomic datasets of breast cancer into multiple age groups and investigated whether a machine learning procedure achieved better performance after the split of age groups.

## Materials and Methods

### Summary of Datasets

This study utilized the transcriptomic and methylomic datasets from The Cancer Genome Atlas database (TCGA) (Ma and Ellis, 2013). The level-3 transcriptomes of the TCGA breast cancer (BRCA) project were hybridized and measured by the Agilent 244K Custom Gene Expression G4502A-07-3 array (TCGA platform code AgilentG4502A_07_3), which was designed by the University of North Carolina on the Agilent (Santa Clara, CA, United States) Sure Print G3 Microarray Platform (Cancer Genome Atlas Network, 2012). Each sample has the expression levels of 17,814 probe sets. The developmental stage of each sample was retrieved from the entry “tumor_stage” in the clinical annotations of the TCGA-BRCA project at the NIH National Cancer Institute GDC Data Portal (Cancer Genome Atlas Network, 2012; Ciriello et al., 2015). There were 502 transcriptomic samples with the stage annotations, among which there were 90, 291, 108, and 13 samples for stages I, II, III, and IV, respectively.

Methylome was generated by the Illumina Infinium HumanMethylation450K BeadChip, and each sample had 485,577 features (Morris and Beck, 2015). There were 765 methylomic samples with the stage annotations in the TCGA-BRCA project, among which there were 125, 433, 196, and 11 samples for stages I, II, III, and IV, respectively.

### Feature Selection Algorithms

Biomedical datasets have two major types, either a large feature number with a small sample number or a large sample number with a small feature number. The OMIC datasets usually extract a large number of features for a small number of samples, and the number of features must be reduced to avoid the overfitting problem for machine learning modeling (Lyu et al., 2017; Ye et al., 2017; Ali and Aittokallio, 2018; Xu et al., 2018). For the second style of biomedical datasets, although it is not a required step, reducing the dimensions may substantially increase modeling performance (Guan et al., 2018; Zou et al., 2018).

Seven feature selection algorithms were evaluated for their classification performances on the datasets with different age groups. The *F*-test evaluated the analysis of variation between two variables, or a variable and the phenotype (Lomax and Hahs-Vaughn, 2013). The PCC (Pearson Correlation Coefficient) was used to evaluate how significantly a feature was associated with the phenotype (Yoon and Chung, 2013). The classic *T*-test was also chosen to rank the features by their association significance with the phenotype (Kim, 2015;Ye et al., 2017).

The Recursive Feature Elimination (*RFE*) strategy was evaluated based on three different algorithms. The Support Vector Machine (SVM) was frequently used to facilitate the procedure of recursive feature elimination and denoted as *rfeSVM* (Xu et al., 2018). The L1 regularization was known as the least absolute shrinkage and selection operator and generated weights for each chosen feature (Guyon and Elisseeff, 2003). The RFE procedure based on Lasso was denoted as rfeLasso (Sfakianakis et al., 2014). The logistic regression (LR) model was also used to calculate how the features were eliminated by their weights (Pandey et al., 2018).

TriVote (Tri-Step Feature Voting algorithm) was recently proposed to perform very well on both transcriptomic and methylomic data and evaluated on the datasets in this study (Xu et al., 2018).

### Classification Algorithms

Classification algorithms may achieve drastically different performances on the same dataset (Ge et al., 2016; Liu et al., 2017; Xu et al., 2018). As a result, in this study, we chose three representative classification algorithms to evaluate the classification performance of a given feature subset, i.e., Logistic Regression (LR), Support Vector Machine (SVM) and Gaussian Naïve Bayes (GaussianNBayes).

Logistic regression calculated the probability of a binary response for a given dataset (Menard, 2018). SVM optimized the maximal separation margin of a discrimination hyperplane between the groups of positive and negative samples, and the discrimination hyperplane tended to have a good binary classification performance (Suthaharan, 2016). The Gaussian Naïve Bayes (GaussianNBayes) assumed the inter-feature independence and calculated the probability that a given query sample belonged to a class (Bouckaert, 2004).

Ten-fold cross-validation was utilized to calculate the binary classification performances (Ren et al., 2018).

### Performance Measurements

A binary classification problem was usually evaluated by the performance metrics accuracy (Acc), sensitivity (Sn), and specificity (Sp) (Xu et al., 2017; Ye et al., 2017). There were two classes of samples in a binary classification problem, denoted as Positive and Negative ones, respectively. There were P and N samples in the classes of Positive and Negative samples. Sensitivity (Sn) was defined as the percentage of correctly predicted positive samples, i.e., Sn = TP/P, where TP (True Positive) was the number of correctly predicted positive samples, and FN (False Negative) was defined as FN = P-TP. The measurement Specificity (Sp) was defined as the percentage of correctly predicted negative samples, i.e., Sp = TN/N, and the number of false positive samples (FP) was defined as FP = N-TN. The overall accuracy was Acc = (TP + TN)/(P + N).

The balanced accuracy [bAcc = (Sn + Sp)/2] was usually utilized to evaluate the classification model without generating bias for a dataset with significantly different numbers of positive and negative samples (Feng et al., 2018). Matthew’s correlation coefficient (MCC) was defined as MCC = (TP × TN-FP × FN)/sqrt[(TP + FP) × (TP + FN) × (TN + FP) × (TN + FN)], where sqrt() is the squared root (Xu et al., 2018; Zhang et al., 2018; Zhao et al., 2018).

### Experimental Design

**Figure 1:**
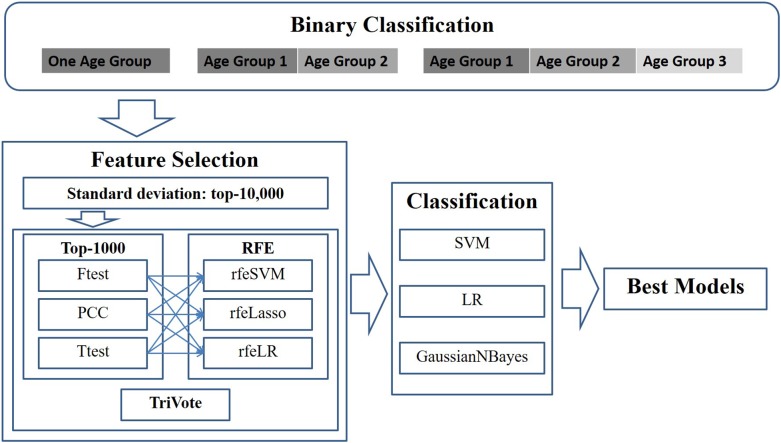
Experimental design of this study. Pairs of three filters and three RFE feature selection algorithms were evaluated for their binary classification performances on the datasets with different age groups. Three binary classifiers were utilized to calculate the classification performances.

This study modeled the early detection of breast cancer as a binary classification problem, due to the fact that there were much fewer samples in stage IV than the other three stages. A binary classification problem was defined as a discrimination function to separate samples between stages I/II and III/IV. The investigations in this study were planned as shown in the outline in Figure 1.

First, a given dataset was screened by variance, which was defined as the average of the squared deviations from the mean in the Python numpy.var(). This study supposed that an OMIC-feature with a large standard deviation may be clinically detected more easily. Thus, this step kept 10,000 features with the largest standard deviations for further biomarker screening.

Then, the dataset was screened by one of the three algorithms (*F*-test, PCC, and *T*-test) for the associations of each feature with the class label. The top 1000 ranked features were kept for further analysis. Iteratively, the remaining dataset was evaluated by one of the recursive feature elimination algorithms (rfeSVM, rfeLasso, and rfeLR), and the feature with the smallest weight was removed from the dataset while the remaining dataset was processed repeatedly. This study decided that the numbers of features would be between 10 and 100 with a step size of 5.

## Results and Discussion

### Data Preprocessing

First of all, we need to rule out the hypothesis that the sample age was correlated with the tumor stages. The Pearson correlation coefficient (PCC) (Mpairaktaris et al., 2017; Zhang et al., 2017) between the sample age and the tumor stage was -0.0221 with *P*-value = 0.6206 for the transcriptome samples. The methylome samples had PCC = -0.0223 with *P*-value = 0.5377 between the sample age and the tumor stage. The hypothesis was rejected for both the transcriptome and methylome samples. The maximal information coefficient (MIC) is very sensitive in detecting weak or non-linear correlations (Reshef et al., 2011) and has been widely used in feature selection (Ge et al., 2016) and inter-gene synergy (Xing et al., 2017), etc. The MIC value was in the range [0, 1] and a larger MIC value means a higher correlation between the two variables. The MIC values between age and tumor stage were 0.0591 and 0.0490 for transcriptome and methylome samples, respectively. These two MIC values were similar to that of the random correlations, as described in Reshef et al. (2011). As a result, both PCC and MIC correlation measurements rejected the hypothesis that the sample age was correlated with the tumor stages.

Among the 502 transcriptomic samples in the TCGA-BRCA project, there were 121 and 381 samples in the early stages (I and II) and late stages (III and IV), respectively. This dataset was denoted as *RNA(1)*. The early-stage patients were regarded as the negative class, and the late-stage ones were the positive class.

Each class of samples was split into two or three bins with equally-sized sample age ranges, as illustrated in Table 1. The minimum age of samples with either transcriptome or methylome was 26, and the maximum age was 90. We used the upper integers of (20 + 70 × *i*/*k*) as the thresholds. The age bins for *k* = 2 were [20, 55) and [55, 90], while the age bins for *k* = 3 were [20, 44), [44, 67), and [67, 90].

**Table 1:** Samples with transcriptomes (RNA) and methylomes (Methy) were grouped using the same age bins.

Age thresholds			[20, 55)	[55, 90]	
*k* = 2	RNA	P	51	70	
		N	153	228	
	Methy	P	93	114	
		N	222	336	

**Age thresholds**			**[20, 44)**	**[44, 67)**	**[67, 90]**

*k* = 3	RNA	P	21	71	29
		N	56	227	98
	Methy	P	31	121	55
		N	67	345	146

*Samples in the early stages (I and II) were denoted as positives, and the other samples in the late stages (III and IV) were the negatives*.

The 121 negative samples were split into two groups with 51 and 70 samples. Moreover, the two groups of positive samples had 153 and 228 members. This dataset was denoted as *RNA(2)*. The two pairs of negative and positive groups were denoted as RNA(2)(0) and RNA(2)(1). The dataset RNA(1) was also split into three bins with equally-sized sample age ranges, which was denoted as *RNA(3)*. The three groups of negative samples in *RNA(3)* had 21, 71 and 29 members, respectively, and the positive class was split into three groups with 56, 227 and 98 members. The three pairs of negative and positive groups were denoted as RNA(3)(0), RNA(3)(1) and RNA(3)(2).

The 765 methylomic samples had 207 early-stage and 558 late-stage samples and were denoted as the dataset *Methy(1)*. The two classes in *Methy(1)* were split into two bins with equally-sized sample age ranges, which was denoted as the dataset *Methy(2)*. There were 93 and 114 members in the two negative groups. The sizes of the two positive groups were 222 and 336. Thus, we had two pairs of negative and positive groups, denoted as Methy(2)(0) and Methy(2)(1). The dataset *Methy(3)* was constructed by splitting the two classes of samples in *Methy(1)* into three bins with equally-sized sample age ranges. There were 31, 121 and 55 members in the three negative groups. The sizes of the three positive groups were 67, 345 and 146. The three pairs of negative and positive groups Methy(3)(0), Methy(3)(1) and Methy(3)(2) refer to the three split datasets.

The 17,814 features were first reduced to the 10,000 with the largest variance, as described in the Section “Materials and Methods.”

### An Initial Investigation of *T*–Test-Selected Features on Transcriptomes

The *T*-test was widely used to evaluate how significantly a feature was associated with the phenotype for various biomedical data types, including transcriptome (Ye et al., 2017), methylome (Aref-Eshghi et al., 2015), imaging data (Beheshti et al., 2016), etc. As described in the above Section “Materials and Methods,” the top 1000 features ranked by the *T*-test were further screened by the three RFE algorithms, i.e., *rfeSVM*, *rfeLasso*, and *rfeLR*.

**Figure 2:**
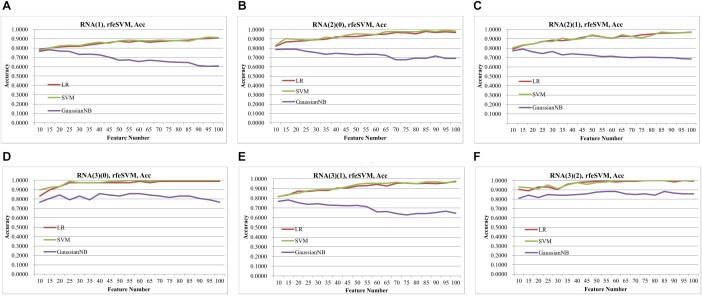
Classification performances of rfeSVM screening of top-ranked 1000 features by *T*-test. The accuracy was calculated by the 10-fold cross validation of three classifiers, i.e., LR, SVM, GaussianNBayes. The horizontal axis was the number of features screened by rfeSVM, and the vertical axis was accuracy. The plots were for the datasets **(A)** RNA(1), **(B)** RNA(2)(0), **(C)** RNA(2)(1), **(D)** RNA(3)(0), **(E)** RNA(3)(1), and **(F)** RNA(3)(2).

Figure 2 demonstrated that the classifier GaussianNBayes did not perform very well on the features screened by rfeSVM. For the first dataset of 10 rfeSVM-screened features, GaussianNBayes (Acc = 0.7629) performed slightly worse than the other two classifiers LR (Acc = 0.7849) and SVM (Acc = 0.7769). When more features were chosen by rfeSVM, GaussianNBayes performed even worse classification. It is interesting to observe that LR and SVM seemed to have performed similarly well. As a result, we generated a more precise summary of how the three classifiers performed, as shown in Table 2. The data suggested that SVM achieved maximal accuracy in 75 cases while LR achieved the same in 39 cases. Unfortunately, GaussianNBayes did not achieve maximal accuracy at any point.

**Table 2:** The number of times each classifier achieved the best accuracy for the RFE-screened features of a given dataset.

rfeSVM	LR	SVM	GaussianNB	rfeLasso	LR	SVM	GaussianNB	rfeLR	LR	SVM	GaussianNB
RNA(1)	6	13	0	RNA(1)	8	11	0	RNA(1)	10	9	0
RNA(2)(0)	3	16	0	RNA(2)(0)	16	2	1	RNA(2)(0)	13	6	0
RNA(2)(1)	10	9	0	RNA(2)(1)	7	11	1	RNA(2)(1)	10	9	0
RNA(3)(0)	4	15	0	RNA(3)(0)	10	9	0	RNA(3)(0)	11	8	0
RNA(3)(1)	6	13	0	RNA(3)(1)	15	4	0	RNA(3)(1)	11	8	0
RNA(3)(2)	10	9	0	RNA(3)(2)	12	7	0	RNA(3)(2)	10	8	1
**Total**	**39**	**75**	**0**	**Total**	**68**	**44**	**2**	**Total**	**65**	**48**	**1**

*There were 19 feature subsets screened by rfeSVM/rfeLasso/rfeLR, with the numbers of features 10, 15, 20, …, 100*.

Table 2 also suggested that GaussianNBayes outperformed the other two classifiers SVM and LR only on very few feature subsets screened by rfeSVM/rfeLasso/rfeLR. For most of the feature subsets chosen by the three RFE algorithms, the two classifiers SVM and LR performed similarly well. We further generated another summary table to demonstrate whether each of the three classifiers achieved the best accuracy across the 19 feature subsets of each dataset, as shown in Table 3. We may observe that the best classifier was usually SVM or LR, and sometimes these two classifiers performed the same best accuracy. Moreover, for all six datasets, rfeSVM outperformed the other two RFE feature selection algorithms. As a result, the following sections would use rfeSVM as the RFE screening choice.

**Table 3:** Summary of whether each classifier achieved the best classification accuracy on the 19 feature subsets of each dataset.

Dataset	RFE	MaxAcc	Classifiers
RNA(1)	**rfeSVM**	**0.9183**	**SVM**
RNA(1)	rfeLasso	0.7669	LR
RNA(1)	rfeLR	0.8725	SVM
RNA(2)(0)	**rfeSVM**	**0.9951**	**SVM**
RNA(2)(0)	rfeLasso	0.8284	SVM
RNA(2)(0)	rfeLR	0.9363	LR, SVM
RNA(2)(1)	**rfeSVM**	**0.9732**	**LR**
RNA(2)(1)	rfeLasso	0.7718	LR
RNA(2)(1)	rfeLR	0.9094	SVM
RNA(3)(0)	**rfeSVM**	**1.0000**	**SVM**
RNA(3)(0)	rfeLasso	0.8961	SVM
RNA(3)(0)	rfeLR	0.9740	LR, SVM
RNA(3)(1)	**rfeSVM**	**0.9732**	**LR**
RNA(3)(1)	rfeLasso	0.7819	LR
RNA(3)(1)	rfeLR	0.9228	SVM
RNA(3)(2)	**rfeSVM**	**1.0000**	**LR, SVM**
RNA(3)(2)	rfeLasso	0.9055	LR
RNA(3)(2)	rfeLR	0.9685	SVM

*Column “MaxAcc” provides the maximal accuracy achieved by the three classifiers on the 19 feature subsets screened by the RFE algorithm given in the Column “RFE”. The column “Classifiers” provides the algorithms achieving the maximal accuracy in the column “MaxAcc.” More than one classifier may achieve the same best accuracy. Best model of each dataset was illustrated in bold*.

### Comparison of *T*-Test, *F*-Test, and PCC for Association Evaluation

A comparison was carried out to evaluate whether the choice of the top 1000 features was important for the binary classification problem of early-stage breast cancer detection, as shown in Table 4. The pair comprised of the feature selection algorithm *F*-test and the classifier SVM achieved the best accuracies for all six datasets. The classifier LR also achieved the same best accuracy for the three datasets RNA(2)(0), RNA(3)(0), and RNA(3)(2). Thus, the default modeling procedure in the following sections started with the top 1000 features ranked by *F*-test. Then, rfeSVM was utilized to find the number of features with the best accuracy calculated by the 10-fold cross-validation of the classifier SVM.

**Table 4:** Summary of whether each classifier achieved the best classification accuracy on the 19 feature subsets of each dataset.

Dataset	FS	MaxAcc	Classifiers
RNA(1)	*T*-test	0.9183	SVM
RNA(1)	***F*-test**	**0.9422**	**SVM**
RNA(1)	PCC	0.9223	SVM
RNA(2)(0)	*T*-test	0.9951	SVM
RNA(2)(0)	***F*-test**	**1.0000**	**LR, SVM**
RNA(2)(0)	PCC	0.9951	SVM
RNA(2)(1)	*T*-test	0.9732	LR
RNA(2)(1)	***F*-test**	**0.9966**	**SVM**
RNA(2)(1)	PCC	0.9765	SVM
RNA(3)(0)	***T*-test**	**1.0000**	**SVM**
RNA(3)(0)	***F*-test**	**1.0000**	**LR, SVM**
RNA(3)(0)	**PCC**	**1.0000**	**SVM**
RNA(3)(1)	*T*-test	0.9732	LR
RNA(3)(1)	***F*-test**	**1.0000**	**SVM**
RNA(3)(1)	PCC	0.9765	SVM
RNA(3)(2)	***T*-test**	**1.0000**	**LR, SVM**
RNA(3)(2)	***F*-test**	**1.0000**	**LR, SVM**
RNA(3)(2)	**PCC**	**1.0000**	**SVM**

*Column “MaxAcc” provides the maximal accuracy achieved by the three classifiers on the 19 feature subsets screened by rfeSVM. The initial subset of 1000 features was ranked by the algorithm given in the Column “FS.” The column “Classifiers” provides the algorithms achieving the maximal accuracy in the column “MaxAcc.” More than one classifier may achieve the same best accuracy. Best model of each dataset was illustrated in bold*.

### Age Grouping for Transcriptomes

**Figure 3:**
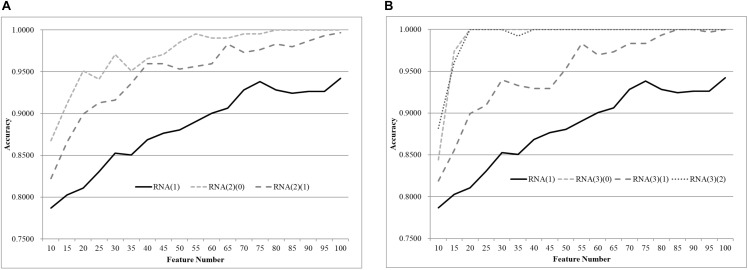
Comparison of early-stage breast cancer detection models with different age groups based on transcriptome data. The patients were split into **(A)** two groups and **(B)** three groups with equally-sized age ranges. The horizontal axis shows the numbers of features chosen by rfeSVM and the vertical axis shows the 10-fold cross validation accuracy of the classifier SVM. *F*-test was used to generate the initial subset of the 1000 top-ranked features.

We first split the negative and positive samples into two equally-sized groups, as shown in Figure 3A. The SVM models trained over RNA(2)(0) and RNA(2)(1) were much better than that on the whole dataset RNA(1). The averaged improvement in accuracy was 0.0900 for the dataset RNA(2)(0) compared to RNA(1). The model accuracy of RNA(2)(1) was also improved by 0.0654 in accuracy on average. If we chose the best model of each dataset as the final result, both RNA(2)(0) and RNA(2)(1) were improved at least 0.0544 in accuracy compared against RNA(1). The best model of RNA(1) used 100 features to achieve 0.9422 in accuracy, while only 40 features were needed for both RNA(2)(0) and RNA(2)(1) to outperform this model.

Similar results were observed for the experiment of splitting RNA(1) into three equally-sized groups of samples, as shown in Figure 2B. The averaged improvements in accuracy were 0.1078, 0.0673, and 0.1086 for the three datasets RNA(3)(0), RNA(3)(1), and RNA(3)(2). A minimum 0.0578 improvement in accuracy was achieved for all three datasets compared with the best model of RNA(1). Only 50 features were required for the three datasets RNA(3)(0), RNA(3)(1), and RNA(3)(2) to outperform the complete dataset RNA(1) (0.9422 in accuracy with 100 features).

### Age Grouping for Methylomes

**Figure 4:**
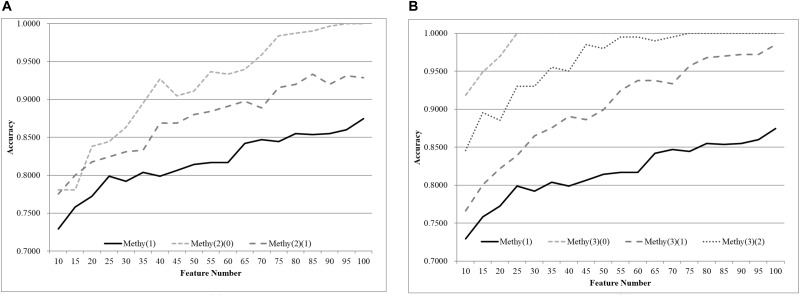
Comparison of early-stage breast cancer detection models with different age groups based on methylome data. The patients were split into **(A)** two groups and **(B)** three groups with equally-sized age ranges. The horizontal axis depicts the numbers of features chosen by rfeSVM and the vertical axis depicts the 10-fold cross-validation accuracy of the classifier SVM. *F*-test was used to generate the initial subset of the 1000 top-ranked features.

The same default classification procedure on the datasets with smaller age groups outperformed that of the complete dataset Methy(1), as shown in Figure 4. A minimum 0.0524 improvement in accuracy was achieved against the complete dataset Methy(1), if the dataset was split into two groups with equally-sized age ranges. The best model for Methy(1) achieved 0.8745 in accuracy with 100 features, while the classifier SVM achieved 0.9910 and 0.9353 in accuracy for the two datasets with smaller age groups, i.e., Methy(2)(0) and Methy(2)(1). Even better improvements were achieved for datasets with smaller age groups. The classifier SVM achieved 1.0000, 0.9958, and 1.0000 in accuracies for the three smaller datasets Methy(3)(0), Methy(3)(1), and Methy(3)(2), respectively. Only 40 features were needed by these three datasets to outperform that of the complete dataset Methy(1).

### TriVote Selected Features for Both Transcriptomes and Methylomes

**Figure 5:**
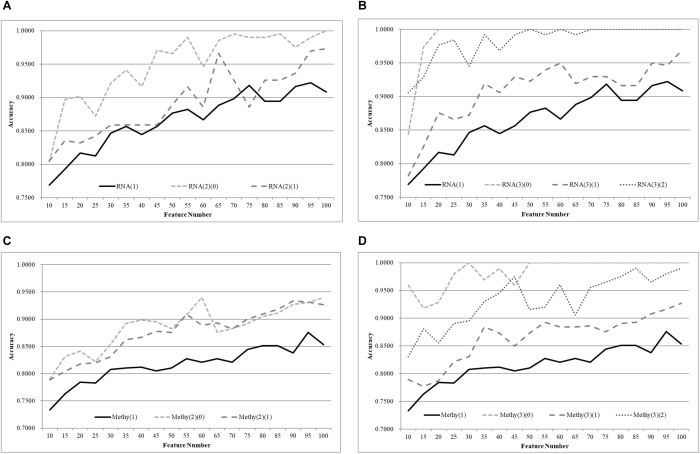
Comparison of early-stage breast cancer detection models with different age groups based on both transcriptome and methylome data. Transcriptomes of the patients were split into **(A)** two groups and **(B)** three groups with equally-sized age ranges. The methylomes were split into **(C)** two groups and **(D)** three groups in the same way. The horizontal axis shows the numbers of features chosen by TriVote and the vertical axis shows the 10-fold cross-validation accuracy of the classifier SVM. *F*-test was used to generate the initial subset of the 1000 top-ranked features.

A comparison between different age groups was also conducted using a recently published feature selection algorithm, TriVote (Xu et al., 2018), as shown in Figure 5. TriVote selected features with very good accuracies on both transcriptomes and methylomes calculated by the best classifier SVM, mentioned above. We have a similar pattern in that a biomedical classification problem may be improved simply by splitting the samples into multiple groups with equally-sized age ranges. The best model on the dataset RNA(1) with the accuracy 0.9223 was achieved by 95 features, as shown in Figure 5, while the two smaller groups RNA(2)(0) and RNA(2)(1) achieved their best accuracies, 0.9412 and 0.9664, with only 35 and 65 features, respectively. Moreover, the best models of both datasets outperformed the best model of RNA(1), with at least 0.0508 in accuracy. An average improvement of 0.0676 was achieved by merely splitting the dataset RNA(1) into three smaller groups with equally-sized age ranges.

Similar patterns were also observed on the TriVote-selected feature subsets, as shown in Figure 5C,D. TriVote achieved average accuracy improvements of 0.0607 and 0.0965 for the cases of two and three groups with equally-sized age ranges.

**Figure 6:**
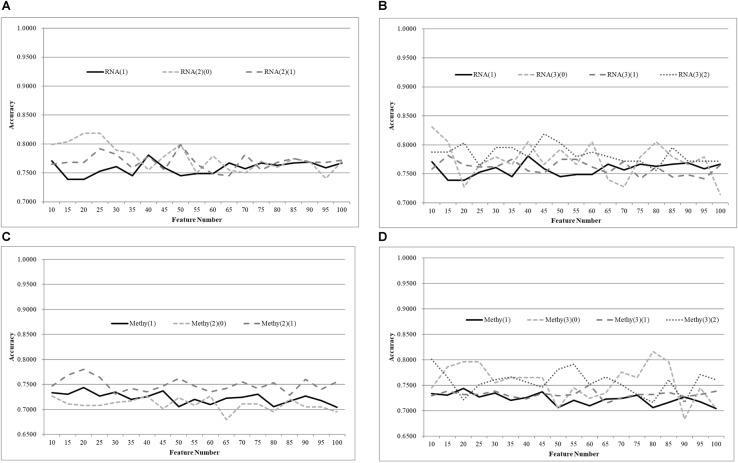
Comparison of early-stage breast cancer detection models with different age groups based on both transcriptome and methylome data. Transcriptomes of the patients were split into **(A)** two groups and **(B)** three groups with equally-sized age ranges. The methylomes were split into **(C)** two groups and **(D)** three groups in the same way. The horizontal axis shows the numbers of features chosen by TriVote and the vertical axis shows the 10-fold cross-validation accuracy of the classifier RFC. *F*-test was used to generate the initial subset of the 1000 top-ranked features.

**Figure 7:**
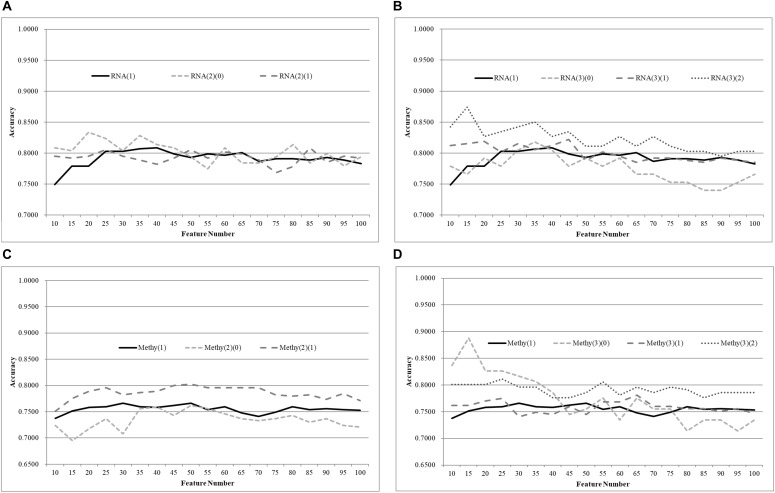
Comparison of early-stage breast cancer detection models with different age groups based on both transcriptome and methylome data. Transcriptomes of the patients were split into **(A)** two groups and **(B)** three groups with equally-sized age ranges. The methylomes were split into **(C)** two groups and **(D)** three groups in the same way. The horizontal axis shows the numbers of features chosen by TriVote and the vertical axis shows the 10-fold cross-validation accuracy of the classifier XGB. *F*-test was used to generate the initial subset of the 1000 top-ranked features.

We further evaluated our hypothesis using two more classifiers, Random Forest Classifier (RFC) (Pal, 2005; Gislason et al., 2006) (Figure 6) and XG boost (XGB) (Chen and Guestrin, 2016) (Figure 7). A similar pattern was observed, but RFC achieved weaker improvements in Acc, as shown in Figure 6. RFC also did not achieve Acc higher than 0.8500. Even weaker improvements in Acc were performed by the age-specific models trained by the classifier XGB, as shown in Figure 7. For example, only 0.0123 and 0.0294 in Acc improvements were achieved by the age-specific XGB models.

### SVM Models on the Independent Test Datasets Using the Features Selected by *F*-Test and rfeSVM

This section covers the investigation of the best algorithms on the independent test sets. Features selected by *F*-test and rfeSVM tended to achieve the best performances, as demonstrated in Figure 3–5. Table 4 suggests that the classifier SVM usually achieved the best classification accuracies. A stratified splitting strategy was used to get 10% of samples as an independent test dataset, which was used for evaluating the model trained over the other samples. The classification performances were iteratively calculated over the next 10% of samples to ensure that all samples were tested.

**Figure 8:**
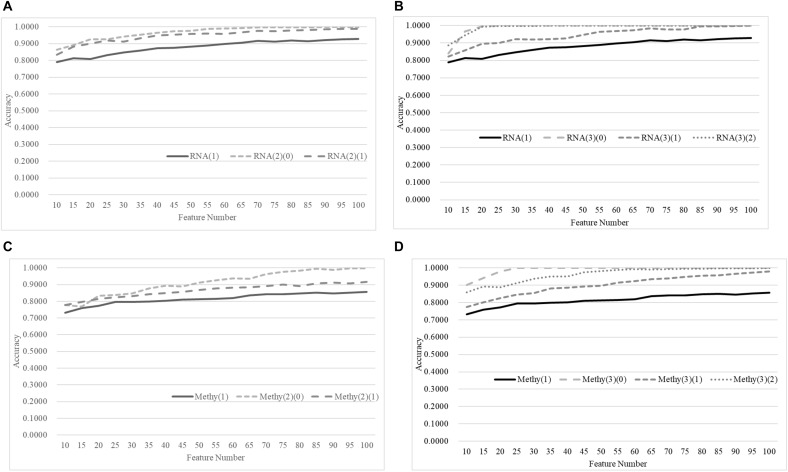
Comparison of early-stage breast cancer detection models with different age groups based on both transcriptome and methylome data using the classifier SVM on the training datasets. Transcriptomes of the patients were split into **(A)** two groups and **(B)** three groups with equally-sized age ranges. The methylomes were split into **(C)** two groups and **(D)** three groups in the same way. The horizontal axis shows the numbers of features chosen by rfeSVM and the vertical axis shows the 10-fold cross-validation accuracy of the classifier SVM. *F*-test was used to generate the initial subset of 1000 top-ranked features.

**Figure 9:**
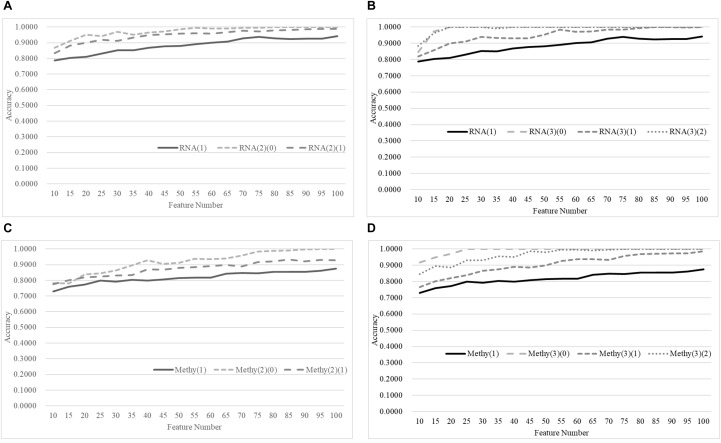
Comparison of early-stage breast cancer detection models with different age groups based on both transcriptome and methylome data using the classifier SVM on the independent test datasets. Transcriptomes of the patients were split into **(A)** two groups and **(B)** three groups with equally-sized age ranges. The methylomes were split into **(C)** two groups and **(D)** three groups in the same way. The horizontal axis shows the numbers of features chosen by rfeSVM, and the vertical axis shows the accuracy of the classifier SVM on the independent test dataset. *F*-test was used to generate the initial subset of 1000 top-ranked features.

Figure 8 demonstrates that the age-specific models outperformed the age-dependent models for both transcriptomes and methylomes on the total dataset, while Figure 9 suggests that a similar relationship was observed between the age-independent models and the age-specific models.

### Comparison of Age-Independent and Age-Specific Models on the Head-Neck Squamous Cell Carcinoma (HNSC) Samples

**Figure 10:**
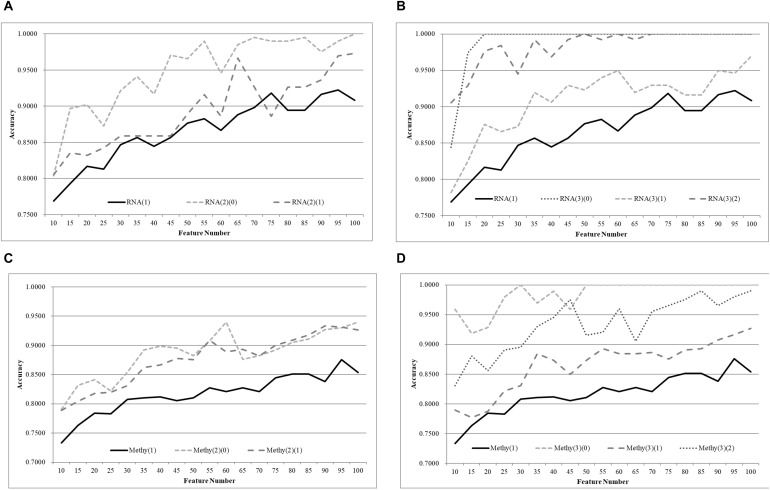
Comparison of early-stage breast cancer detection models with different age groups based on both transcriptome and methylome data using the classifier SVM on the dataset TCGA-HNSC. Transcriptomes of the patients were split into **(A)** two groups and **(B)** three groups with equally-sized age ranges. The methylomes were split into **(C)** two groups and **(D)** three groups in the same way. The horizontal axis shows the numbers of features chosen by rfeSVM and the vertical axis shows the 10-fold cross-validation accuracy of the classifier SVM on the independent test dataset. *F*-test was used to generate the initial subset of the 1000 top-ranked features.

We further analyzed the TCGA-HNSC (Head-Neck Squamous Cell Carcinoma) dataset for our hypothesis to see whether the age-specific models outperformed the age-independent ones, as shown in Figure 10. The analysis procedure with the best performance was utilized for the TCGA-HNSC dataset, i.e., the SVM classifier on the *F*-test + rfeSVM feature selection duet.

The age-independent model in the solid lines in Figure 10 demonstrated very good accuracies (Acc = 0.9223 for transcriptome and Acc = 0.8758 for methylome). However, at least a 0.05 improvement in Acc may be achieved by building two age-specific transcriptome models, as in Figure 10A. The averaged improvement 0.0676 in Acc may be achieved if the transcriptome dataset is split into three age groups, as in Figure 10B. The classification accuracy of the age-independent methylome model may be improved by 0.0607 and 0.0965 on average for the two-group and three-group age-specific models, respectively (Figure 10C,D).

## Conclusion

This study carried out a series of extensive modeling experiments and demonstrated that age was an essential factor in selecting biomarkers. A biomarker-based disease diagnosis model may be improved by simply splitting the samples into multiple groups with smaller age ranges. SVM achieved the largest Acc improvements compared with the other classification algorithms. It should be further investigated how age could be directly integrated into the biomarker selection and diagnosis modeling.

We have tried to investigate the discrimination model between cancer and control samples. Unfortunately, there only 1 transcriptome and 6 methylome control samples contained both stage and age data, respectively. These sample numbers were much fewer than those of the cancer samples. We regret that we did not find the dataset to compare cancer and normal samples with our proposed age-specific models.

## Data Availability

Publicly available datasets were analyzed in this study. This data can be found here: https://portal.gdc.cancer.gov/projects/TCGA-BRCA.

## Author Contributions

FZ and XF conceived the project and designed the experiments. XF, JL, HL, HC, FL, and QL wrote the codes and conducted the experiments. XF, JL, FL, and QL generated the experimental results and drafted the discussions. FZ and Z-HY discussed the experimental design and polished the manuscript. FZ and XF drafted and polished the manuscript.

## Conflict of Interest Statement

The authors declare that the research was conducted in the absence of any commercial or financial relationships that could be construed as a potential conflict of interest.
